# The Effect of Season and Weather on Orthopaedic Trauma: Consult Volume Is Significantly Correlated with Daily Weather

**DOI:** 10.1155/2018/6057357

**Published:** 2018-09-02

**Authors:** J. M. Wilson, C. A. Staley, A. L. Boden, A. R. Boissonneault, A. M. Schwartz, M. L. Schenker

**Affiliations:** ^1^Department of Orthopaedic Surgery, Emory University School of Medicine, Atlanta, Georgia, USA; ^2^Emory University School of Medicine, Atlanta, Georgia, USA

## Abstract

**Introduction:**

On-call orthopedic clinicians have long speculated that daily consult volume is closely correlated with weather. While prior studies have demonstrated a relationship between weather and certain fracture types, the effect of weather on total orthopaedic consult volume has not yet been examined. The aim of this study was to investigate this relationship.

**Methods:**

We retrospectively reviewed orthopaedic consult data from 405 consecutive days at an urban, level one trauma center. The number, mechanism of injury, and type of consult were collected, along with daily weather data (temperature, wind, and precipitation). Statistical analysis was then performed to determine the relationship between weather and orthopaedic trauma consults.

**Results:**

A total of 4543 consults were received during the study period. There was a significant difference in total number of consults between months of the year (p<0.001). A post hoc analysis revealed that this was due to increased volume in the summer months relative to the winter months (i.e., August 13.7 consults/day; January 9.3 consults/day). Average daily temperature and consult volume were also positively correlated (p<0.001, r= 0.30). While there was no significant association between precipitation and total consult volume, when there was over 0.25 inches of rain, there were less penetrating trauma (p=0.034) and motorcycle collision consults (p=0.013).

**Conclusion:**

Weather parameters, specifically average temperature and precipitation, were found to be associated with daily orthopedic consult type and volume. Additionally, consult volume varies significantly between months of the year. Because trauma centers are often resource scarce, this is an important relationship to understand for proper resource allocation.

## 1. Introduction

Clinicians have long speculated that daily trauma volume is closely correlated with the weather, and to many familiar with the care of the traumatically injured this seems anecdotally obvious. Not surprisingly, this conjecture has been the subject of numerous studies, primarily examining the number of general surgery trauma admissions and emergent cases as it relates to daily weather conditions [1–13]. Orthopedic injuries have also received attention in this regard, as a number of studies have examined the relationship between weather and fracture-specific patterns—most notably hip, forearm, and distal radius fractures [14–24].

Given the medical morbidity and mortality associated with hip fractures in the geriatric population, [25] hip fractures have understandably been of particular interest. Because ground level falls are a common mechanism of injury, multiple studies have demonstrated a higher incidence of hip fractures in the winter months [16, 21, 24, 26]. A similar trend has been demonstrated in distal radius fractures [20, 23]. Pediatric orthopedic injuries, on the other hand, have been shown to increase with warm temperatures and decrease with significant precipitation [15]. Unfortunately, the current literature on adult orthopaedic trauma is limited to only a handful of specific fracture types. To our knowledge, only one study has examined the effect of weather metrics on orthopedic consult volume and this was in a pediatric population [15]; however, no similar study exists in adults.

At publicly-funded, urban, level I trauma centers, there are limited resources available, despite the inherently high demands of often severely injured patients. These centers treat a large number of patients and orthopedic trauma typically accounts for a significant portion of this volume. The strain of this large number of resource-intensive patients on the healthcare system becomes problematic, as constraints on operating room availability can increase time to surgery for urgent cases [27]. Examining only a few specific fracture types, while useful, does not fully elucidate the strain that increased volume places on a hospital's staffing and resources. Additionally, severity of injury has been something that prior studies have had difficulty capturing. This study examined severity in two ways: (1) for patients who required a trauma activation, Injury Severity Scores (ISS) were collected from the trauma registry and (2) the number of orthopaedic cases booked as urgent or emergent was examined.

The current study sought to delineate what effect various weather variables, in addition to day of the week, month, and season, have on the total orthopedic trauma volume seen at an American College of Surgeons (ACS)-verified urban, level I trauma center. We hypothesized that orthopedic trauma consult volume as well injury severity in these patients is significantly affected by these parameters. This study aims to provide useful information for optimal resource allocation—consult and operating room coverage, implant availability, and operating room block time. Proper resource allocation could conceivably optimize care of patients from the time they arrive to the trauma center, through definitive care.

## 2. Materials and Methods

This is an IRB approved, retrospective review of orthopedic consults at an ACS-verified urban, level one trauma center in the southeastern United States. Orthopaedic consults were queried for 405 consecutive days ranging from December 2015 to January 2017. Hand and spine call at our institution is shared with plastic surgery and neurosurgery, respectively, so these consults were excluded from analysis. Additionally, the on-call resident covers orthopedic consults for a local pediatric hospital and these were also excluded. Therefore, the patient population included patients over the age of 18 years who required an orthopedic trauma consult.

The following metrics were collected for each of the 405 days: total number of inpatient consults and outpatient consults, total number of combined consults, age, admitting service, injury or reason for consult, and number of urgent/emergent consults. Mechanism of injury [motor vehicle crash (MVC), motorcycle crash (MCC), ground level fall (GLF), gunshot wound (GSW), fall from height (FFH), pedestrian versus auto (PVA), and other blunt mechanism)], Injury Severity Score (ISS), and Glasgow Coma Score (GCS) were queried from the local trauma database for all inpatient consults. This information is available for all patients who required a trauma activation. Given the volume of patients included in this series, this information was collected for only the inpatient consults, because outpatient consults were less likely to require trauma activation and we assumed these patients were not severely injured.

For the purposes of this study, urgent or emergent consults were defined as one of the following: open fractures (ballistic injuries were not included in this cohort), compartment syndrome, septic joint, and traumatic arthrotomy.

Inpatient consults included those patients who arrived to the emergency department (ED) and were admitted, as well as those patients who are already admitted to the hospital with a subsequent orthopedic injury or concern prompting consultation. Outpatient consults were those patients who were evaluated in the emergency department, treated by the orthopedic service, and discharged home with follow-up. Fracture types were classified into the following broad categories: foot (talus, midfoot, metatarsal, and phalanx), calcaneus, femur (shaft and distal), hip (including all femoral neck and peritrochanteric fractures), acetabulum, pelvic ring, tibia (plateau and shaft), pilon, ankle, humerus, both bone forearm fractures, and distal radius fractures. Patients who were multiply injured were tabulated as one overall consult, but each injury was also tabulated independently for categorical analysis.

Weather metrics were gathered from the National Climatic Data Center (NCDC) online databases (https://www.ncdc.noaa.gov). Weather data was collected at a nearby weather station, located 9.3 miles from the institution where the consults were received and well within the catchment range of the hospital. The following weather metrics were collected: precipitation amount, snow amount, average, maximum, and minimum temperature, average wind speed, and fastest 5 seconds of wind speed (when available).

Statistical analysis was completed using Stata (StataCorp. 2015. Stata Statistical Software: Release 14. College Station, TX: StataCorp LP) software. Analysis of variance (ANOVA), independent samples t-tests, and Pearson correlation were then performed to determine the relationship between weather and orthopaedic trauma consults. Findings were considered significant when p<0.05. Where appropriate, correlation coefficients as well as odds ratios are reported.

## 3. Results

### 3.1. Consult Characteristics

During the 405-day study period, there were a total of 5,725 orthopedic consults. Once hand (715 consults) and spine (467 consults) were excluded, 4543 orthopedic trauma consults met inclusion criteria and were included in this study. This equates to an average of 0.46 consults per hour and 11.21 consults per day. Of these, 2874 (63%) were inpatient consults and 1669 (37%) were outpatient consults. Ultimately, 2055 (71.55%) of the inpatient consults required trauma activation and therefore were available for further evaluation. Specific injuries included foot fractures (693 consults), calcaneus fractures (126 consults), femur fractures (74 consults), hip fractures (269 consults), tibia fractures (530 consults), pilon fractures (79 consults), both bone forearm fractures (57 consults), humerus fractures (301 consults), distal radius fractures (291 consults), and ankle fractures (524 consults). The remaining consults did not fit into a specified injury category. There were 503 emergent or urgent consults—382 open fractures, 50 septic joints, 13 cases of compartments syndrome, and 58 traumatic arthrotomies (Table 1).

### 3.2. Consult Variation with Month of Year

**Figure 1:**
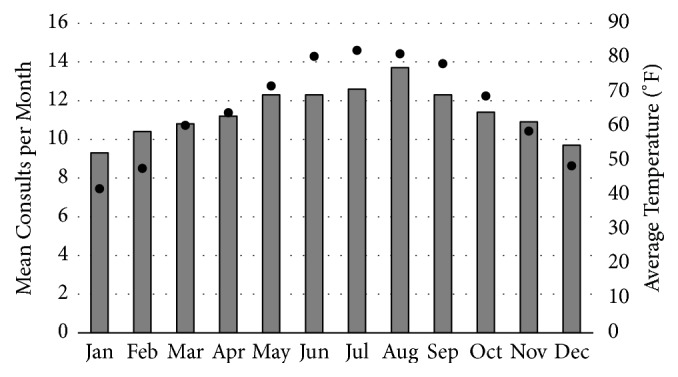
Average number of consults per month and average daily temperature per month (scatter plot). The average daily number of consults increases with increasing average monthly temperature.

A one-way between-subjects ANOVA was conducted to compare the effects of the month of the year on the total number of orthopaedic consults. There was a significant increase in the number of consults in the summer months of the year, with August demonstrating peak consult volume (p<0.001; see Figure 1). A Bonferroni post hoc analysis of pairwise differences revealed that this was due to increased volume in summer months when compared to winter months. Specifically, the following were found to be significant: December versus July (p=0.046) and August (p<0.001); January versus May (p=0.009), June (p=0.009), July (p=0.001), August (p<0.001), and September (p=0.012); February versus August (p=0.020). No other pairwise differences were significant (Figure 1).

### 3.3. Consult Variation with Day of the Week

ANOVA analysis was also utilized to attempt to identify any variations in consult volume by day of the week (Monday, Tuesday, Wednesday, etc.). This analysis revealed no statistically significant variation between days of the week or between weekdays and weekends.

### 3.4. Consult Volume Variation with Temperature

**Figure 2:**
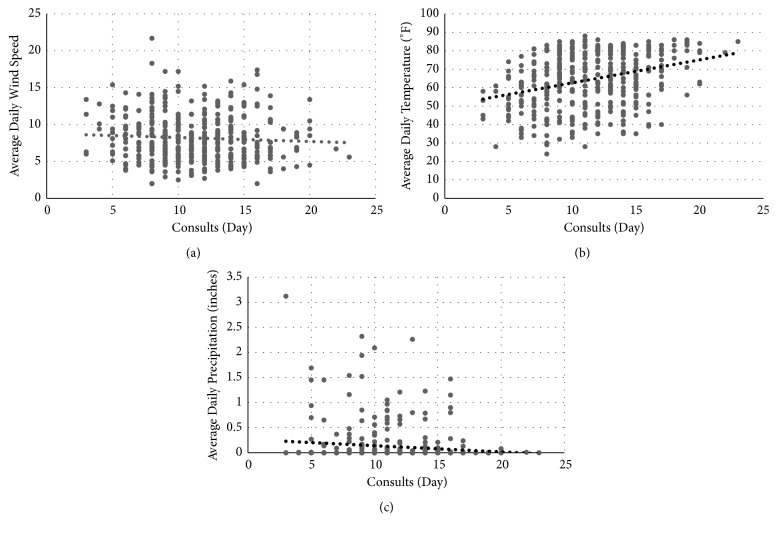
Relationship between total orthopedic consults and weather parameters: (a) average daily wind speed, (b) average daily temperature (r=0.30, p<0.001), and (c) average daily precipitation. Average daily wind speed and average daily precipitation were not significantly correlated to consult volume.

Pearson correlation was utilized to compare total orthopaedic consult volume to daily temperature. There was a significant positive correlation between average daily temperature and total orthopaedic consult volume (r=0.30, p<0.001; see Figure 2). Furthermore, subgroup analysis with Pearson correlation revealed weak, but significant, correlation between average daily temperature and lower extremity trauma (foot, ankle, and pilon; r=0.15, p=0.002), motorcycle crash mechanism (r=0.19, p<0.001), and total blunt trauma (r=0.20, p<0.001). Temperature did not significantly correlate with any other mechanism of injury, nor with any other injury types (Figure 2).

### 3.5. Mechanism of Injury/Consult Volume with Total Precipitation

Independent sample t-tests were used to determine the influence of precipitation on consults. There was no significant association between daily precipitation and total consult volume (p = 0.407; Figure 2(c)). However, when there was over a quarter inch of rain in a single day, there were significantly less penetrating trauma consults (p=0.034) and significantly less motorcycle collisions (p=0.013). Pearson correlation was also utilized to further delineate the relationship between total daily precipitation and consult type and mechanism, and there was no correlation with total consult volume. There was a weak, but significant, negative correlation between precipitation and motorcycle collision consults (r = -0.12, p=0.013; see Table 2). There was also a trend toward a significant negative correlation between total daily precipitation and consults from gunshot wounds (r= -0.091; p= 0.067; Table 2).

### 3.6. Mechanism of Injury/Consult Volume and Wind Speed

Pearson correlation revealed no significant correlation between wind speed (maximum 5-second gust or average daily wind speed) and consult volume (p = 0.776). There were no significant correlations found between wind speed and types of consult or mechanism of consult. (Table 2, Figure 2)

### 3.7. Severity of Injury and Weather

Weather parameters did not significantly correlate with ISS scores or urgent/emergent consult volume. Consult Injury Severity Score was not correlated with average daily temperature (p=0.514), total precipitation (p=0.704), or wind speed (p=0.809). Similarly, open fracture consults were not associated with temperature (p=0.410), precipitation (p= 0.415), or wind (p=0.352). There were also no significant correlations found with traumatic arthrotomies, septic joints, or compartment syndromes.

## 4. Discussion

Weather having an effect on human behavior and injury is not a novel concept [28]. In his 1842 “thermic law of delinquency,” Adolphe Quetelet hypothesized that violent crimes were more prevalent in the summer months. While there have been multiple reports on the incidence of specific fracture types and total pediatric orthopaedic consult volume, [1–8, 17] with regards to weather, there has been, to our knowledge, no report examining total adult orthopedic trauma consult volume as it relates to various weather metrics. Most studies have concluded that, in the pediatric population, total trauma admissions and fracture incidences are increased in the spring and summer. The opposite has been found in geriatric populations where hip fracture rates are increased during winter months.

In the present study, we examined the effect of various weather parameters on all orthopedic trauma consult volume. Our data demonstrated that consult volume was positively correlated with temperature and, as such, there were significantly more daily consults in summer months when compared with winter months. Adverse weather (precipitation, wind, and snow) was not found to have a significant effect on total consult volume. However, when precipitation was greater than 0.25 inches there was significantly less penetrating and motorcycle crash consult volume. There was no difference in severity of injury or the number of urgent/emergent consults with variation in weather parameters. Our results also did not demonstrate a significant difference in consult volume by day of the week, a finding that was anecdotally surprising.

A strength of our study is that data was analyzed from over 400 consecutive days. The study was performed at a high volume, level one trauma center, with weather data available from the National Weather Service in close proximity to the treating hospital. Additionally, all data was from one center and daily consult data was compared directly to daily observed weather. We were also able to investigate the effect of weather on consult severity by comparing average consult ISS and daily number of urgent/emergent consults to weather variables.

The retrospective nature of our design limits the conclusions that can be drawn, and the generalizability of our results may be limited. Hospitals in areas with a similar climate to the southeastern United States are likely to experience similar patterns to those demonstrated in our study. However, more northern climates, with significantly lower temperatures and higher amounts of winter precipitation, may experience different seasonal variation in total consult volume, given the wider ranges of both temperature and precipitation between winter and summer months. Additionally, it must be noted that this study was conducted at a high-volume, level one trauma center in a major metropolitan area. Generalizing the results to smaller, nonlevel one centers outside of a metropolitan area is of unknown appropriateness. Lastly, our database only allows for identification of diagnosis at time of consult. Therefore, we likely underestimate the incidence of both septic joint and compartment syndrome as the workup may have been pending when reported or the condition may have yet to develop in the case of compartment syndrome.

Seasonal variation in consult volume and subsequently case volume is an important relationship to understand. The emergency department literature has examined volume variation and utilizes this information to appropriately allocate bed and staffing resources [29–31]. In orthopedics, this would be a novel concept but one that makes good sense. Implant availability, resident and faculty call coverage, and operating room block time availability should be properly allocated to match local seasonal variation needs. Orthopedic trauma surgeons would likely benefit from understanding their local variation and reserve more operating block time for trauma in the “busy season” and schedule elective cases intentionally at slower times of the year.

## 5. Conclusion

In conclusion, our study has demonstrated that total orthopedic trauma consult volume is positively correlated with temperature and this translates to higher orthopedic trauma volume in the summer months when compared to winter months. This variation should be anticipated by similar centers and efforts should be made to optimize patient care through proper resource appropriation.

## Figures and Tables

**Table 1:** Number and type of consults over study period.

Consults	Number of consults
Total	4543
Inpatient	2874
Outpatient	1669
Trauma Activation	2055
Urgent/Emergent	503

Foot Fractures	693
Calcaneus	126
Ankle	524
Both Bone Forearm	57
Femur	374
Hip	269
Acetabulum	193
Pelvic Ring	294
Tibia	530
Pilon	59
Humerus	301
Distal Radius	291

Total Urgent/Emergent Consults	503
Open Fractures	382
Traumatic Arthrotomy	58
Septic Joints	50
Compartment Syndrome	13

**Table 2:** Pearson Correlation analysis of weather parameters versus consult type and mechanism of injury. Statistically significant values are in bold.

**Type of Consult**	v. Temperature	v. Wind	v. Total Precipitation
Total Consults	**r=0.30, p<0.001**	r=-0.01, p=0.776	r=-0.04, p=0.407
Lower Extremity (Foot, ankle, pilon)	**r=0.15, p=0.002**	r=0.01, p=0.764	r=<-0.01, p=0.999
Long Bone (Tibia, Femur)	r=0.01, =0.922	r=-0.03, p=0.508	r=-0.05, p=0.373
Pelvis (hip, acetabulum, pelvic ring)	r=-0.002, p=0.967	r=<0.01, p=0.989	r=0.03, p=0.595
Upper Extremity (BBFFx, DR, Humerus)	r=0.03, p=0.584	r<-0.01, p=0.993	r=-0.03, p=0.565

**Mechanism of Injury**			

GSW	r=0.04, p=0.381	r=-0.03, p=0.514	r=-0.091, p=0.067
MVC	r=0.06, p=0.215	r=0.06, p=0.257	r=0.02, p=0.687
MCC	**r=0.19, p<0.001**	r=0.02, p=0.665	**r=-0.12, p=0.013**
Total Penetrating	r=0.04, p=0.385	r=-0.02, p=0.664	r=-0.09, p=0.063
Total Blunt	**r=0.20, p<0.001**	r=0.04, p=0.386	r=-0.072, p=0.146

## Data Availability

The consult data used to support the findings of this study are restricted by the Emory University IRB in order to protect patient privacy. The weather data used in this study is publicly available and can be found at https://www.ncdc.noaa.gov/cdo-web/.
